# Supplementary material for: White matter alterations in glaucoma and monocular blindness differ outside the visual system
Source: Sci Rep. 2021 Mar 25;11:6866. doi: 10.1038/s41598-021-85602-x (PMC7994383; doi:10.1038/s41598-021-85602-x)
Supplement: Supplementary file 1 — Supplementary Information. [file 41598_2021_85602_MOESM1_ESM.docx]

SUPPLEMENTARY INFORMATION

White matter alterations in glaucoma and monocular blindness differ outside the visual system

**Authors:**

Sandra Hanekamp^1,2,3,*^, Branislava Ćurčić-Blake^4^, Bradley Caron^5,6^, Brent McPherson^1^, Anneleen Timmer^7^, Doety Prins^7^, Christine C. Boucard^8^, Masaki Yoshida^8^, Masahiro Ida^9^, David Hunt^1^, Nomdo M. Jansonius^7^, Franco Pestilli^1,2,3,4#^, and Frans W. Cornelissen^7#^

**Author affiliation:**

^1^Department of Psychological and Brain Sciences, Indiana University, Bloomington, IN, USA

^2^Department of Intelligent Systems Engineering, Luddy School of Informatics and Engineering, Indiana University, Bloomington, IN, USA

^3^ Department of Psychology, The University of Texas at Austin, Austin, TX, USA

^4^ Department of Biomedical Sciences of Cells and Systems, University of Groningen, University Medical Center Groningen, the Netherlands

^5^ Program in Neuroscience, Indiana University, Bloomington, IN, USA

^6^ Program in Vision Science, School of Optometry, Indiana University, Bloomington, IN, USA

^7^ Laboratory for Experimental Ophthalmology, University Medical Center Groningen, University of Groningen, the Netherlands

^8^ Department of Ophthalmology, Jikei University School of Medicine, Tokyo, Japan

^9^ Department of Radiology, National Hospital Organization Mito Medical Center, Ibaraki, Japan

# These authors share senior authorship.

# Abstract

The degree to which glaucoma has effects in the brain beyond the eye and the visual pathways is unclear. To clarify this, we investigated white matter microstructure (WMM) alterations in 37 tracts of patients with glaucoma, monocular blindness, and controls. We used brainlife.io for reproducibility. White matter tracts were subdivided into seven categories ranging from those primarily involved in vision (the visual white matter) to those primarily involved in cognition and motor control. In the vision tracts, WMM was decreased as measured by fractional anisotropy in both glaucoma and monocular blind subjects compared to controls, suggesting neurodegeneration due to reduced sensory inputs. A test-retest approach was used to validate these results. The pattern of results was different in monocular blind subjects, where WMM properties increased outside the visual white matter as compared to controls. This pattern of results suggests that whereas in the monocular blind loss of visual input might promote white matter reorganization outside of the early visual system, such reorganization might be reduced or absent in glaucoma. The results provide indirect evidence that in glaucoma unknown factors might limit the reorganization as seen in other patient groups following visual loss. [193/200]

**Correspondence:** Franco Pestilli [pestilli@utexas.edu](mailto:pestilli@utexas.edu) and Sandra Hanekamp [sandrahanekamp@gmail.com](mailto:sandrahanekamp@gmail.com)

**Supplementary Methods
Inclusion and exclusion criteria of two cohorts**

**Dutch cohort**

Healthy subjects

- Both eyes have an intact visual field. Outcome of Frequency Doubling Technology (C20-1 screenings mode): all test locations are intact (P≥1%).
- A good visual acuity in both eyes. Outcome of the Snellen visual acuity: at least 0.8 (0.1 logMAR or less).

Exclusion:

- Diagnosis with neurological or psychiatric disorders.
- An eye disease.
- Pregnancy.
- Having metal implants or other metal objects in the body.
- Known with claustrophobia .

*Monocular glaucoma subjects*

Inclusion

- In one eye a visual field defect due to primary open angle glaucoma, pseudoexfoliation syndrome or pigment dispersion syndrome.
- The contralateral eye has an intact visual field. Outcome of the Humphrey Field Analyzer (30-2 SITA): glaucoma hemifield test ‘within normal limits’.
- The contralateral eye has a good visual acuity. Outcome of the Snellen visual acuity: at least 0.8 (0.1 logMAR or less).

Exclusion

- Another eye disease than primary open angle glaucoma, pseudoexfoliation syndrome or pigment dispersion syndrome.
- Pregnancy.
- Having metal implants or other metal objects in the body.
- Known with claustrophobia.
- Diagnosis with neurological or psychiatric disorders.

*Monocular blind subjects*

Inclusion

- Unilaterally light-perception negative for at least five years, due to perforation, enucleation, evisceration or ablatio retinae leading to blindness to the affected eye.
- Healthy had to have an intact visual field. Outcome of Frequency Doubling Technology (C20-1 screening mode): all test locations are intact (P≥1%).
- A good visual acuity in the healthy eye. Outcome of the Snellen visual acuity: at least 0.8 (0.1 logMAR or less).

Exclusion

- Existence of monocular vision shorter than 5 years.
- Visus lower than 0.8 in the healthy eye. Outcome of the Snellen visual acuity: at least 0.8 (0.1 logMAR or less).
- Diagnosis with neurological or psychiatric disorders.
- An eye disease.
- Pregnancy.
- Having metal implants or other metal objects in the body.
- Known with claustrophobia .

**Japanese cohort**

*Glaucoma subjects*

Inclusion

- Optic nerve head changes consistent with glaucoma assessed with slit-lamp examination, evaluated by an ophthalmologist
- Optic nerve head changes consistent with glaucoma assessed with Optical Coherence Tomography (OCT), evaluated by an ophthalmologist
- Visual field defects consistent with glaucoma assessed with a Humphrey Field Analyser (HFA), evaluated by an ophthalmologist
- Topically controlled intraocular pressure (IOP) of >21 mmHg.

Exclusion criteria

- Diagnosis with neurological or psychiatric disorders.

*Healthy subjects*

Exclusion criteria
Diagnosis with neurological or psychiatric disorders.

- An eye disease.

**Supplementary Table S1.** Average effect sizes on the white matter microstructure for fractional

anisotropy and mean diffusivity measured across all 37 WM tracts.

|  | **Fractional Anisotropy** | | **Mean Diffusivity** | |
| --- | --- | --- | --- | --- |
| *Comparison* | *Mean (g)* | *Std Error* | *Mean (g)* | *Std Error* |
| **GL1 vs HC1** | -0.25 | 0.04 | 0.09 | 0.04 |
| **GL2 vs HC2** | -0.28 | 0.04 | 0.20 | 0.04 |
| **MBL vs HC1** | 0.28 | 0.04 | -0.37 | 0.04 |
| Average effect sizes for each group comparison were calculated based on all white matter tracts. Between group differences were determined using Two-Way (Factorial) ANOVA was used independently for the two glaucoma datasets and respective control groups. The average effect size per group comparison revealed that the effect of glaucoma and monocular blindness on the WM microstructure differed significantly between groups (both FA and MD F<0.0001). | | | | |

| **Supplementary Table S2.** Categorical effect size on the white matter microstructure based on the effect size of fractional anisotropy and mean diffusivity. | | | | | | | |
| --- | --- | --- | --- | --- | --- | --- | --- |
| **Category** | **WM Tracts** | **Fractional Anisotropy** | | | **Mean Diffusivity** | | |
|  |  | *JP*  *GL vs HC* | *NL*  *GL vs HC* | *NL*  *MBL vs HC* | *JP*  *GL vs HC* | *NL  GL vs HC* | *NL  MBL vs HC* |
| **Early vision** | OR L | -0.549 | -0.718 | -0.632 | 0.082 | 0.583 | 0.182 |
|  | OR R | -0.912 | -0.697 | -0.372 | 0.727 | 0.023 | -0.135 |
| **Ventral stream** | IFOF L | -0.824 | -0.62 | 0.771 | 0.424 | 0.111 | -0.831 |
|  | IFOF R | -0.808 | -0.29 | 0.252 | 0.566 | 0.168 | -0.123 |
|  | ILF L | -0.761 | -0.72 | -0.111 | 0.361 | 0.28 | -0.332 |
|  | ILF R | -0.89 | -0.725 | -0.182 | 0.421 | 0.284 | -0.093 |
| **Dorsal stream** | Cingulum L | 0.279 | -0.129 | 0.659 | -0.19 | -0.143 | -0.201 |
|  | Cingulum R | -0.078 | -0.105 | 0.421 | 0.137 | -0.119 | -0.279 |
|  | SLF1And2 L | -0.342 | -0.269 | 0.494 | 0.306 | 0.183 | -0.564 |
|  | SLF1And2 R | -0.64 | -0.331 | 0.38 | 0.598 | 0.344 | -0.423 |
|  | SLF3 L | -0.191 | -0.586 | 0.509 | 0.286 | 0.132 | -0.607 |
|  | SLF3 R | -0.197 | -0.428 | 0.452 | 0.25 | 0.269 | -0.456 |
| **Occipital** | Forceps Major | -0.19 | -0.145 | 0.209 | 0.307 | -0.247 | -0.165 |
|  | VOF L | -0.139 | -0.368 | -0.172 | 0.526 | 0.172 | -0.078 |
|  | VOF R | 0.097 | -0.895 | -0.181 | 0.22 | 0.403 | -0.09 |
| **Vertical** | MDLFang L | -0.069 | 0.015 | 0.448 | 0.303 | 0.263 | -0.22 |
|  | MDLFang R | 0.104 | -0.135 | 0.347 | 0.441 | -0.013 | -0.319 |
|  | MDLFspl L | 0.033 | 0.006 | 0.166 | 0.115 | -0.115 | -0.403 |
|  | MDLFspl R | -0.184 | -0.555 | 0.218 | 0.089 | 0.182 | -0.434 |
|  | pArc L | -0.311 | -0.244 | 0.45 | 0.35 | 0.235 | -0.357 |
|  | pArc R | -0.167 | -0.163 | 0.403 | 0.214 | 0.253 | -0.372 |
|  | TPC L | 0.09 | -0.373 | 0.13 | 0.021 | 0.172 | -0.311 |
|  | TPC R | -0.049 | -0.295 | 0.185 | 0.169 | 0.107 | -0.272 |
| **Callosal** | CC AnterioFrontal | -0.207 | 0.061 | 0.478 | -0.009 | -0.399 | -0.411 |
|  | CC MiddleFrontal | -0.01 | -0.022 | 0.593 | -0.196 | -0.044 | -0.632 |
|  | CC Parietal | -0.72 | -0.027 | 0.28 | 0.748 | -0.096 | -0.285 |
|  | Forceps Minor | -0.292 | -0.18 | 0.318 | -0.071 | -0.316 | -0.318 |
| **Other** | Arcuate L | -0.519 | -0.084 | 0.619 | 0.147 | 0.156 | -0.509 |
|  | Arcuate R | -0.398 | -0.213 | 0.515 | 0.329 | 0.232 | -0.464 |
|  | Aslant L | -0.363 | -0.048 | 0.354 | 0.108 | 0.107 | -0.466 |
|  | Aslant R | 0.001 | -0.27 | 0.094 | 0.015 | 0.281 | -0.354 |
|  | ATR L | -0.102 | 0.363 | 0.521 | -0.087 | -0.316 | -0.6 |
|  | ATR R | 0.025 | 0.095 | 0.603 | -0.233 | -0.114 | -0.5 |
|  | CST L | -0.185 | -0.281 | 0.2 | -0.006 | 0.096 | -0.707 |
|  | CST R | -0.152 | 0.225 | 0.24 | 0.106 | -0.05 | -0.477 |
|  | Uncinate L | -0.362 | -0.248 | 0.292 | -0.223 | 0.064 | -0.502 |
|  | Uncinate R | -0.342 | 0.068 | 0.556 | 0.027 | 0.199 | -0.619 |
| The effect of glaucoma and monocular blindness on the white matter microstructure compared to healthy controls per category and group. Positive effects sizes indicate that the value of the clinical group (either FA or MD) was increased compared to the healthy controls. | | | | | | | |

**Supplementary Table S3. Number of subjects with excluded white matter tract**

| **Tract** | *GL_JP* | *HC_JP* | *GL_NL* | *HC_NL* | *MBL_NL* |
| --- | --- | --- | --- | --- | --- |
| **L OR** | 5 | 3 | 2 | 1 | 2 |
| **R OR** | 3 | 3 | 3 | 1 | 2 |
| **Forceps Minor** | 0 | 0 | 0 | 2 | 0 |
| **Forceps Major** | 0 | 0 | 0 | 2 | 0 |
| **Parietal CC** | 1 | 0 | 1 | 2 | 0 |
| **Middle Frontal CC** | 0 | 0 | 0 | 2 | 0 |
| **Anterior Frontal CC** | 0 | 0 | 0 | 2 | 0 |
| **L Cingulum** | 3 | 0 | 1 | 4 | 2 |
| **R Cingulum** | 0 | 0 | 1 | 3 | 0 |
| **L Uncinate** | 0 | 0 | 1 | 2 | 2 |
| **R Uncinate** | 0 | 0 | 0 | 2 | 0 |
| **L IFOF** | 5 | 2 | 6 | 8 | 6 |
| **R IFOF** | 4 | 1 | 0 | 3 | 1 |
| **L Arcuate** | 1 | 0 | 0 | 2 | 0 |
| **R Arcuate** | 3 | 0 | 0 | 2 | 0 |
| **L SLF 1/2** | 0 | 0 | 1 | 2 | 0 |
| **R SLF 1/2** | 0 | 0 | 0 | 2 | 0 |
| **L SLF 3** | 0 | 0 | 0 | 2 | 0 |
| **R SLF 3** | 0 | 0 | 0 | 2 | 0 |
| **L Fronto Thalamic** | 1 | 0 | 0 | 2 | 0 |
| **R Fronto Thalamic** | 1 | 0 | 0 | 2 | 0 |
| **L Aslant** | 1 | 0 | 0 | 2 | 0 |
| **R Aslant** | 1 | 0 | 0 | 3 | 0 |
| **L ILF** | 0 | 0 | 0 | 2 | 0 |
| **R ILF** | 0 | 0 | 0 | 2 | 0 |
| **L MDLF Ang** | 1 | 0 | 0 | 2 | 0 |
| **R MDLF Ang** | 1 | 1 | 0 | 2 | 0 |
| **L MDLF SPL** | 5 | 3 | 4 | 2 | 1 |
| **R MDLF SPL** | 3 | 4 | 3 | 3 | 2 |
| **L pArc** | 0 | 0 | 0 | 2 | 0 |
| **R pArc** | 1 | 0 | 0 | 2 | 0 |
| **L TPC** | 2 | 0 | 0 | 2 | 0 |
| **R TPC** | 2 | 0 | 0 | 2 | 0 |
| **L VOF** | 0 | 1 | 0 | 2 | 0 |
| **R VOF** | 0 | 0 | 0 | 2 | 0 |
| **L CST** | 1 | 0 | 0 | 2 | 0 |
| **R CST** | 1 | 0 | 0 | 2 | 0 |
| Number of subjects with specific tracts excluded in each dataset. Tracts were excluded if they had less than 50 streamlines. | | | | | |

**Supplementary analysis 1. Are the results robust to the subjects selection criteria?**

For exploratory purposes, we repeated our analysis with two different subsets of our Japanese glaucoma cohort (GL2). The objective of this exploratory analysis was to examine whether a more homogenous selection of subjects would result in a similar effect on white matter (WM) tract categories as observed in our main analysis. To achieve a more homogenous group of glaucoma subjects, we performed analysis with a subset of subjects that had a visual field defect (VFMD) with a mean deviation greater than – 2 dB in the ‘Better Eye’. These subjects could be considered monocular glaucoma (n=12). Next, we also examined the effect on the remaining subjects that could be considered having binocular glaucoma as they had a VFMD smaller than – 2 dB in the “Better Eye”. This resulted in a subset of GL2 of 18 subjects. The effect size was calculated for fractional anisotropy (FA) between groups using the average tract values of our 37 WM tracts. In addition to our main analysis, we compared GL2; subset monocular versus HC2 and GL2; subset binocular versus HC2. The Effect sizes were computed using Hedges’ G for each tract using The Measures of Effect Size (MES) Toolbox ([github.com/hhentschke/measures-of-effect-size-toolbox](http://www.github.com/hhentschke/measures-of-effect-size-toolbox)). Next, the resulting Hedges’ G value for each tract was averaged per tract category to create an average effect size measure of WM property changes. For more details see **Methods: statistical analyses.** To examine whether the effect of the different glaucoma subsets was similar between groups, we investigated the main effect of groups for Hedges’ g FA using a Two-Way Factorial ANOVA. The main effect of groups consisted of three levels: (1) GL2 Original versus HC2, (2) GL2 Monocular subset versus HC2, (3) GL2 Binocular subset versus HC. No significant effect between groups was found F(13,1)=0.07, p<0.80. To examine whether WM tract categories were differently affected between groups, we examined the interaction effect between groups and white matter categories using Two-Way Factorial ANOVA. We did not find a significant effect F(13,6)=1.96, p<0.09. Average effect sizes per groups can be found in **Supplementary Table S4** and **Supplementary** **Figure S1** and indicate that although differences appear, the overall pattern remains consistent between different subsets.

| **Supplementary Table S4.** Average effect sizes on the white matter microstructure for fractional anisotropy measured across white matter tract categories. | | | | | | | |
| --- | --- | --- | --- | --- | --- | --- | --- |
| **Comparison** | **Early vision** | **Ventral stream** | **Occipital** | **Dorsal stream** | **Callosal** | **Vertical** | **Other** |
| **MBL vs HC1** | -0.5 | 0.18 | -0.05 | 0.49 | 0.42 | 0.29 | 0.4 |
| **GL1 vs HC1** | -0.71 | -0.59 | -0.47 | -0.31 | -0.04 | -0.22 | -0.04 |
| **GL2 vs HC2** | -0.73 | -0.82 | -0.08 | -0.19 | -0.31 | -0.07 | -0.24 |
| **GL2 vs HC2  >2dB; Monocular** | -0.45 | -0.81 | -0.24 | -0.43 | -0.5 | -0.15 | -0.23 |
| **GL2 vs HC2  >2dB; Binocular** | -1.08 | -0.93 | -0.06 | -0.05 | -0.22 | -0.07 | -0.28 |

| 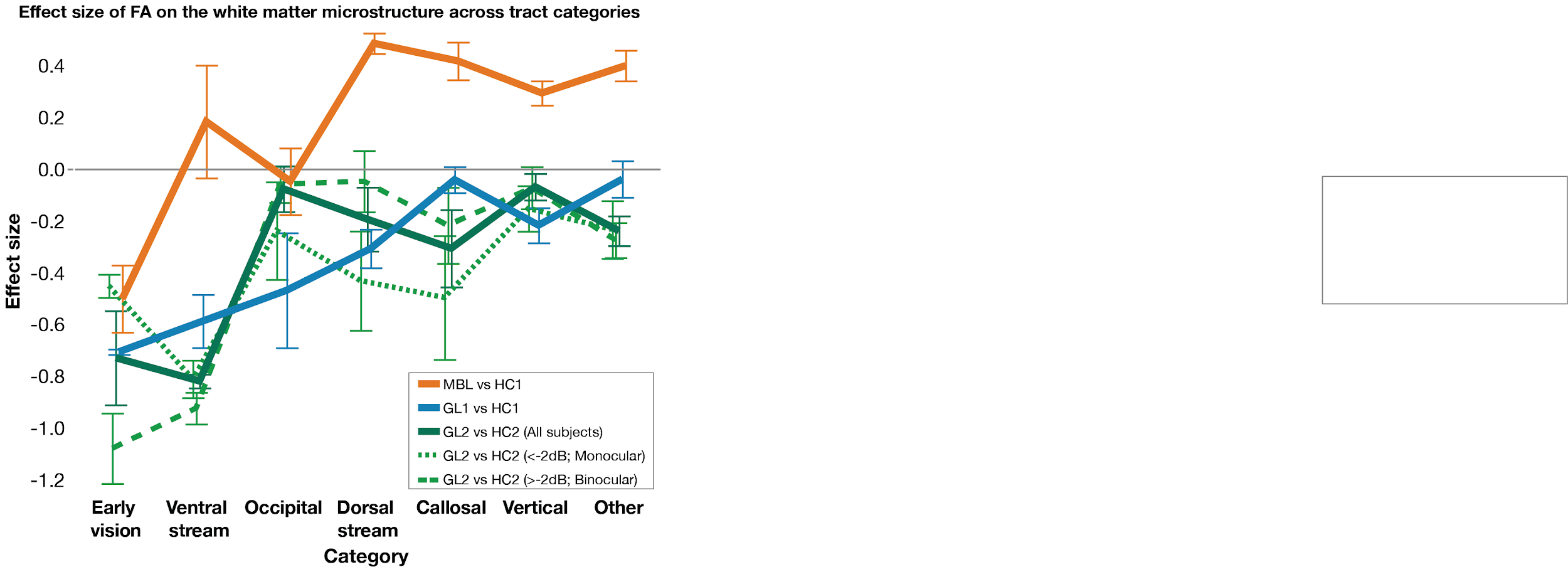 |
| --- |
| **Supplementary Figure S1. Effect size of FA on the white matter microstructure across tract categories.** The effect of glaucoma and monocular blindness on the WM microstructure compared to healthy controls is visualized for each tract category (abscissa) and participant groups (colors). Positive effects sizes indicate that the microstructural estimate FA was increased in the clinical group as compared to the healthy controls. |

|  |
| --- |
| 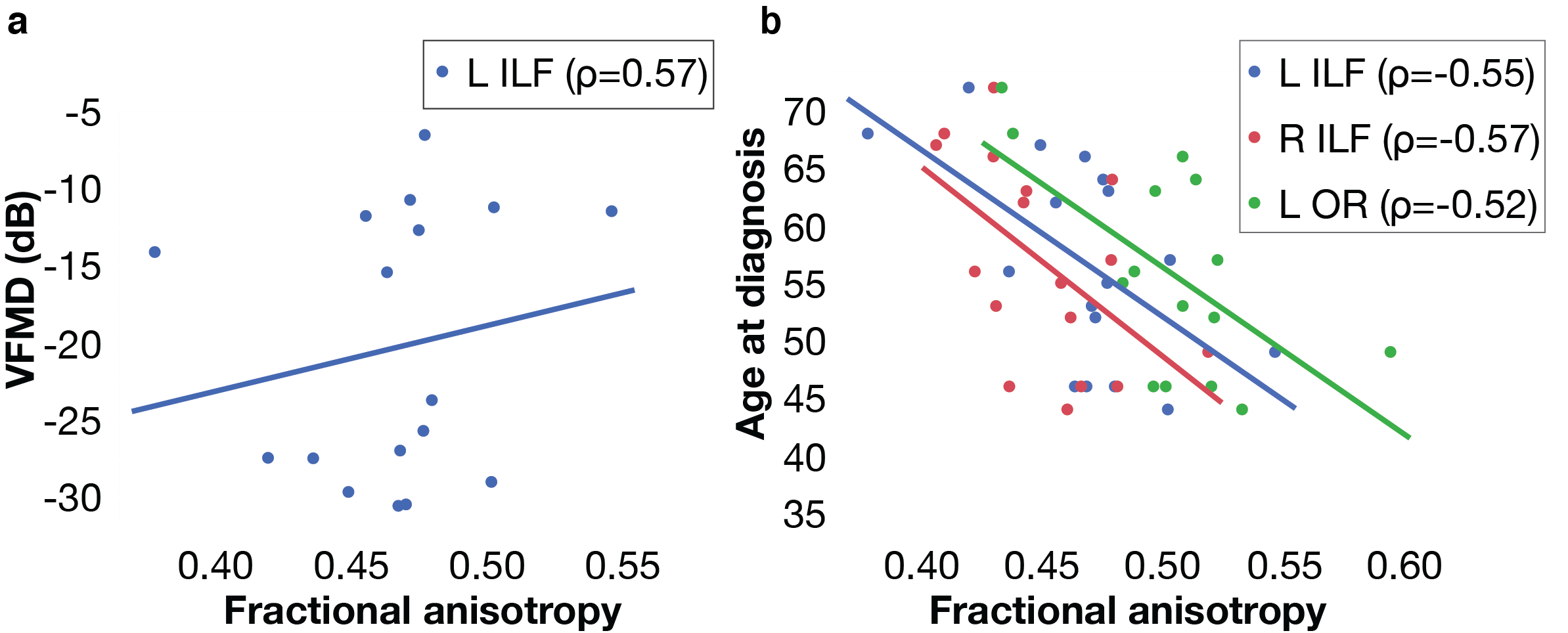 |
| **Supplementary Figure S2.** **Correlations between clinical features and fractional anisotropy in the Dutch glaucoma subjects (GL1).** The scatterplot shows the correlation between the clinical characteristics of glaucoma patients of the Dutch cohort and fractional anisotropy values of white matter tracts. VFMD= Visual Field Mean Deviation (dB), ILF= inferior longitudinal fasciculus, OR=optic radiation, R=right hemisphere, L= left hemisphere. |

**Supplementary information - List of Abbreviations**

| AD | Axial Diffusivity |
| --- | --- |
| Ang | Angular |
| Arc | Arcuate Fasciculus |
| ATR | Anterior Thalamic Radiation |
| CC | Corpus Callosum |
| Cing | Cingulum Cingulate |
| CST | Corticospinal Tract |
| DWI | Diffusion-weighted Magnetic Resonance Imaging |
| FA | Fractional Anisotropy |
| FAT | Frontal Aslant Tract |
| FMaj | Forceps Major |
| FMin | Forceps Minor |
| GL | Glaucoma |
| GMWMI | Grey Matter White Matter Interface |
| HC | Healthy Control |
| IFOF | Inferior Frontal Longitudinal Fasciculus |
| ILF | Inferior Longitudinal Fasciculus |
| JP | Japanese |
| L | Left |
| MBL | Monocular Blindness |
| MD | Mean Diffusivity |
| MLF | Medial Longitudinal Fasciculus |
| MRI | magnetic resonance imaging |
| NL | Netherlands |
| NTG | Normal Tension Glaucoma |
| OR | Optic Radiation |
| pArc | Parietal Arcuate Fasciculus |
| R | Right |
| RD | Radial Diffusivity |
| SLF | Superior Longitudinal Fasciculus |
| SPL | Superior Parietal Lobule |
| TPC | Temporal Parietal Connection |
| UF | Uncinate Fasciculus |
| VOF | Ventral Occipital Fasciculus |
| VFMD | Visual Field Defect Mean Deviation |
| WM | White Matter |

# 
